# Workplace gender-based violence and associated factors among university women in Enugu, South-East Nigeria: an institutional-based cross-sectional study

**DOI:** 10.1186/s12905-021-01273-w

**Published:** 2021-03-23

**Authors:** Olaoluwa Samson Agbaje, Chinenye Kalu Arua, Joshua Emeka Umeifekwem, Prince Christian Iheanachor Umoke, Chima Charles Igbokwe, Tochi Emmanuel Iwuagwu, Cylia Nkechi Iweama, Eyuche Lawretta Ozoemena, Edith N. Obande-Ogbuinya

**Affiliations:** 1 Department of Human Kinetics and Health Education, Faculty of Education, University of Nigeria, Nsukka, Nigeria; 2 Department of Physical and Health Education, Faculty of Education, Alex-Ekwueme Federal University, Ndufu-Alike, Ebonyi State Nigeria

**Keywords:** Gender-based violence, Workplace bullying, Incivility, Sexual harassment, University women, Nigeria

## Abstract

**Background:**

Exposure to workplace gender-based violence (GBV) can affect women's mental and physical health and work productivity in higher educational settings. Therefore, this study aimed to examine the prevalence of GBV (workplace incivility, bullying, sexual harassment), and associated factors among Nigerian university women.

**Methods:**

The study was an institutional-based cross-sectional survey. The multi-stage sampling technique was used to select 339 female staff from public and private universities in Enugu, south-east Nigeria. Data was collected using the Workplace Incivility Scale (WIS), Modified Workplace Incivility Scale (MWIS), Negative Acts Questionnaire-Revised (NAQ-R), and Sexual Experiences Questionnaire (SEQ). Descriptive statistics, independent samples *t*-test, Pearson’s Chi-square test, univariate ANOVA, bivariate, and multivariable logistic regression analyses were conducted at 0.05 level of significance.

**Results:**

The prevalence of workplace incivility, bullying, and sexual harassment (SH) was 63.8%, 53.5%, and 40.5%. The 12-month experience of the supervisor, coworker, and instigated incivilities was 67.4%, 58.8%, and 52.8%, respectively. Also, 47.5% of the participants initiated personal bullying, 62.5% experienced work-related bullying, and 42.2% experienced physical bullying. The 12-month experience of gender harassment, unwanted sexual attention, and sexual coercion were 36.5%, 25.6%, and 26.6%, respectively. Being aged 35–49 years (AOR 0.15; 95% CI (0.06, 0.40), and ≥ 50 years (AOR 0.04; 95% CI (0.01, 0.14) were associated with workplace incivility among female staff. Having a temporary appointment (AOR 7.79, 95% CI (2.26, 26.91) and casual/contract employment status (AOR 29.93, 95% CI (4.57, 192.2) were reported to be associated with workplace bullying. Having a doctoral degree (AOR 3.57, 95% CI (1.24, 10.34), temporary appointment (AOR 91.26, 95% CI (14.27, 583.4) and casual/contract employment status (AOR 73.81, 95% CI (7.26, 750.78) were associated with workplace SH.

**Conclusions:**

The prevalence of GBV was high. There is an urgent need for workplace interventions to eliminate different forms of GBV and address associated factors to reduce the adverse mental, physical, and social health outcomes among university women.

**Supplementary Information:**

The online version contains supplementary material available at 10.1186/s12905-021-01273-w.

## Background

Gender-based violence (GBV), or violence against women in the workplace is a major public health problem globally. The World Bank’s Inter-Agency Standing Committee defines GBV as "an umbrella term for any harmful act that is perpetrated against a person's will, and that is based on socially ascribed (gender) differences between males and females” [1]. Furthermore, GBV has been conceptualized as violence towards minority groups, individuals, and/or communities solely based on their gender, which can directly or indirectly result in psychological, physical, and sexual traumas or injury and deprivation of their right as a human being [2]. GBV primarily involves violence against a person based on gender (i.e., both men and women) [1–3]; however, women bear the brunt of violence due to the prevailing gender inequalities [4]. For instance, epidemiological studies [4–7] reported that GBV undermines the daily life activities of women.

In Nigeria, the prevalence of GBV is high. Previous studies reported that GBV is an important public health problem in Nigeria [4, 8–10]. For instance, a study [8] reported that about 52.1% of the women indicated that domestic violence incidence is high, while 63.3% had experienced domestic violence at one time or the other. Sexual abuse was the most frequently reported form of abuse experienced [10]. The high prevalence of GBV in Nigeria has been attributed to a culture of silence, cultural values, and practices [4, 8].

Also, research evidence suggests that GBV has deleterious effects on women’s health. Such adverse health outcomes include physical injuries, mental health problems, sexual and reproductive health problems, sexually transmitted infections (STIs), gynecological disorders, poor pregnancy outcomes, poor health outcomes in children of affected women [3, 11].

Previous studies have indicated that GBV is a predominant phenomenon in higher educational institutions [9, 12]. However, this problem is still under-studied in educational institutions in developing nations [13]. Thus, there may be a paucity of data available on the prevalence of GBV among university women in Nigeria. In the present study, we use the concept of GBV to encompass the most common and potential forms of workplace violence against women in higher education systems, such as the university environment. A previous study [14] adopted this approach. However, the present study focused on incivility, bullying, and sexual harassment among female university staff.

Workplace incivility refers to a subtle form of negative interpersonal behavior characterized by rudeness and disrespect [15, 16]. Incivility also implies rude speech or behavior, impoliteness, bad manners, and inappropriateness [17]. From the victim's view, workplace incivility is caused by individuals such as coworkers/colleagues, clients or supervisors who exhibit rude behaviors towards him or her. Similarly, incivility exemplifies uncivil behavior that has low-intensity and that the intention to harm is not apparent [16]. Leiter [18] posited the uncivil workplace behaviors could be an integral part of an organization's climate or culture rather than as an individual phenomenon. Regardless of its subtleness, incivility has been considered as a risk factor for more severe aggressive behavior and adverse health outcomes [19]. In numerous work settings, women are more likely than men to experience uncivil behaviors such as rude and discourteous comments, and men are the primary perpetrators of workplace incivility [16, 20, 21]. Examples of uncivil behaviors in the workplace include receiving a commendation for others' endeavors, peddling unverified reports about coworkers, nonchalant attitude towards collective tasks, sending unwanted emails to colleagues [19, 22].

Previous studies affirm that incivility can precipitate many adverse outcomes in the workplace, including university setting. Workplace incivility can result in academic stress, poor motivation, and low productivity, and absenteeism [23], mental health problems [16, 19], low self-efficacy [24], poor self-control [25], diminished task performance [26], and burnout [27].

Moreover, incivility has been identified to be closely linked with other forms of workplace GBV such as bullying, abuse, harassment, antisocial behavior, and social undermining [27, 28]. Workplace bullying (WPB) is a prevalent public health problem in many regions of the world [29]. Einarsen et al. [30] conceptualized workplace bullying as an act of harassing, offending, socially excluding someone, or negatively affecting someone's work tasks. Similarly, for an activity to be termed bullying, it has to be perpetrated repeatedly and regularly and over some time (e.g., about six months). Additionally, workplace bullying refers to repeated hurtful detrimental acts or acts (physical, verbal, or psychological intimidation) involving criticism and humiliation to cause fear, distress, or harm to the individual [31].

The two major linked forms of WPB identified in extant literature include work-related bullying (i.e., unfair deadlines, insurmountable workloads, excessive monitoring, and a feeling of denial of access to relevant information), and personal bullying. Personal bullying includes the persistent experience of gossip, discourteous/rude comments, unwarranted teasing, and persistent criticism [32]. Also, many factors besides individual factors (i.e., inadequate social competencies and psychosomatic symptoms) have been identified to promote and trigger WPB's perpetuation in diverse organizational and cultural climes. Such factors include power distance, uncertainty avoidance, fear of employee to express disagreement, patriarchalism, the overall decision-making process [33], organizational culture and climate [34], working conditions and job design [35], leadership [36], role conflict and role ambiguity [37]. Akella [31] further asserted that communities characterized by high power distance and low in uncertainty avoidance support the occurrence of workplace bullying.

A plethora of studies have identified the adverse outcomes of WPB [30, 38, 39]. For instance, WPB creates a toxic environment [40] with adverse outcomes such as diminished corporate/organizational productivity, decreased work motivation, a lack of concentration, errors, and absenteeism [41, 42], sleep disorders, anxiety, chronic fatigue, anger, depression, and several somatic disorders and decreased performance [30, 43]. Women in academia may be more prone to WPB than other work contexts due to high-stress levels [44]. Thus, identifying the prevalence of WPB among female university staff could offer profound insights that may further inform appropriate interventions.

Furthermore, exposure to workplace incivility and bullying could also lead to workplace sexual harassment (WSH). Workplace SH has been identified as a severe public health problem in extant literature. Sexual harassment is any form of unwanted verbal, non-verbal or physical conduct of a sexual nature that occurs with the purpose or effect of violating a person's dignity, particularly when creating an intimidating, hostile, degrading, humiliating or offensive environment [45]. Also, WSH is a form of workplace harassment typically characterized by gender or sex lines [46]. Besides, the literature suggests that women are more likely than men to experience sexual harassment in a lifetime [47–49]. Fitzgerald identified three dimensions of SH. These include gender harassment (GH), unwanted sexual attention (UwSA), and sexual attention (SA). Gender harassment entails verbal and nonverbal behaviors that portray abusive, unfriendly, or undignified attitudes towards women. GH's primary purpose is not sexual intercourse; however, it accentuates the dispersion of attitudes that foster hatred of women.

In contrast, UwSA encompasses forms of sexual advances perceived by the victim as offensive, unwanted, and unrequited. Such can include requests for dates, letters, phone calls, touching, grabbing, and other sexual assaults forms. Sexual coercion highlights the request for sexual favors as compensation for job rewards or prospects.

Previous studies [9, 50–52] have reported a high prevalence of SH in higher educational settings. Also, prior studies [50, 52] had reported that women in most cases are the victims of SH in higher educational settings. Women exposed to SH in the workplace experience adverse health outcomes such as decreased job satisfaction, long-term sickness absence, depression, and anxiety [53–56]. The literature further shows that SH negatively impacts the victims' mental health [53, 56]. Since SH is a preventable occupational health problem, concerted efforts are needed to identify its magnitude and predictors in the Nigerian university context. Thus, the present study is birthed as part of the efforts to ascertain the prevalence of SH and associated factors among university women in south-east Nigeria.

Research evidence has shown that an interplay of different factors influences GBV perpetration and victimization. For instance, past studies [57–59] identified age, rural residence, parity, childhood exposure or experience of violence, educational status, marital conflict, partner, and personal substance use as the predictors of GBV. GBV, as a complex and multidimensional concept, is influenced by an interplay of several factors, such as personal, situational, and sociocultural factors [60]. This understanding supports the underlying assumptions of the social ecological model (SEM). Therefore, we employed the socio-ecological model to investigate associated factors of GBV among university women, such as individual and institutional. The SEM posits that multiple factors interact to influence health behaviors and efforts designed to motivate an individual to change their behavior should embrace all the factors or web of influence that support such behaviors to be effective. The SEM identifies the individual as the core of an ecosystem and offers a valuable and integrative framework to enhance an in-depth understanding of the numerous factors that sustain systemic perpetuation of GBV in higher education systems and those that hinder its eradication [61, 62]. The socio-ecological models [61, 62] provide a wide-ranging framework of systems and interactive levels such as intrapersonal, interpersonal, institutional, community, and policy that helped explain the associated factors of GBV perpetration, victimization and further informs interventions that can be implemented at each level to address GBV.

This study aimed to determine whether the prevalence of incivility, bullying and sexual harassment (i.e., forms of GBV) is high among university women and examines if women's GBV experiences are associated with their personal factors and contextual variables (staff category, employment status). Next, we hypothesized that there are interrelationships among the outcomes-workplace bullying, incivility, and sexual harassment. Hopefully, the findings may substantiate and add to the existing data on the prevalence of GBV and associated factors among university women. This study may further increase an understanding of factors that influence GBV perpetration and contribute to prevention programs. The findings can also help identify evidence-based prevention interventions and those for mitigating the effects of GBV exposure among university women.

## Methods

### Study design and setting

This study was an institutional-based cross-sectional design. It was conducted in Enugu, south-east Nigeria. The study period covered five months from May 25 to October 30, 2019. The Igbo communities mainly inhabit Enugu state. People from other tribes also reside in the states. Examples of such tribes include Yoruba, Hausas/Fulani, Itsekiri people, Ibibio and Efik people, Idoma people, Igala people, etc. Enugu state has a population of 3,267,837 people, according to the 2006 population census [63]. The University of Nigeria Enugu Campus (UNEC) is a federal tertiary institution in Enugu city. Also, the Enugu State University of Science and Technology (ESUT) is a state university located in Enugu and Agbani, respectively. Private/mission universities such as Renaissance University with its main campus in Ugbawka, Enugu; Godfrey Okoye University, Enugu; Caritas University, Amorji-Nike, Enugu. The universities serve as academic hubs for the south-east, south-south, south-west, and the northern states. The population for the study comprises 4995 female staff in the sampled universities during the 2018/2019 academic session. Female employees constitute the bulk of manpower in these universities.

### Sample size determination and procedure

We used the Leslie Kish single population proportion formula to calculate the study sample size. We assumed the prevalence of workplace incivility, bullying, and sexual harassment to be 30% among female university staff with a 95% confidence level and 5% margin of error. Also, a 5% non-response rate was added to the initial sample size. Thus, 339 women constituted the study sample size.

The calculated sample size for the study was 323. Afterwards, the sample size was multiplied by 5% non-response rate (323 * 0.05 = 16) and was added to 323 (i.e., 323 + 16). Finally, the study sample was determined to be 339. The sample size is an approximation. Thus, three hundred and thirty-nine female staff were recruited from the universities in Enugu, Enugu State. Multi-stage random sampling was used to select participants for the study. At the first stage, we stratified the universities to private and public institutions, and subsequently, we randomly selected four out of six universities in Enugu City. Two public and two private universities were selected. In the second stage, a systematic sampling technique was employed to select the faculties using the list of faculties in the respective universities as a sampling frame. The principal investigators and well-trained research assistants approached the eligible participants individually, invited them to participate, and the study’s aims were explained to them. The participants were informed that participation is voluntary and that they can withdraw from participation at any time they deem fit without any reason. When necessary, we provided clarification, and participants were assured that their responses would be treated confidentially and without identity disclosure. We obtained informed verbal consent from the participants. The approval of the University of Nigeria's institutional review board (IRB), Nsukka, was obtained (Reference number: NHREC/05/01/2008B-FWA00002458-IRB00002323). The inclusion criteria include working for at least 12 months as university staff, absence of ill health, and issuance of voluntary informed consent. Exclusion criteria include a work experience of fewer than 12 months, and refusal to participate in the study, and ill-health. Interviews were conducted face-to-face, and each interview lasted, on average, 30–45 min.

### Measures

After obtaining informed verbal consent from the participants, the investigators and trained data collectors administered the demographic information sheet, 7-item workplace incivility Scale (WIS), the 7-item modified workplace incivility Scale (MWIS) by Blau and Andersson, the Negative Acts Questionnaire-Revised (NAQ-R), and the Sexual Experiences Questionnaire (SEQ). The WIS, MWIS, NAQ-R and SEQ are not under license. They are available in the public domain. Thus, licenses were required for their use.

#### Sociodemographic characteristics

Information on demographic characteristics of the participants was collected using an information sheet developed by the researchers. The information sheet collected data on the participant’s age, academic qualification, marital status (having a partner or spouse, divorced, single, widowed), employment status, work experience (i.e., years of experience working as an academic or non-academic staff) salary grade, and staff category/position. Moreover, we coded the participants' age in years, both as a continuous and discrete variable. Participants’ age was categorized as follows: 18–34 years coded as 1; 35–49 years coded as 2; and ≥ 50 years coded as 3 (older female staff). Academic qualification was categorized into five groups such as Senior Secondary School Certificate of Examination-SSCE (coded as 1), Ordinary National Diploma/National Certificate of Examination-OND/NCE (coded as 2), first degree-B.Sc., B.Ed., B.A, etc. (coded as 3), having master’s degree-M.Sc., M.A., M.Ed. (coded as 4). Furthermore, possession of a doctoral degree/Ph.D. (coded as 5). Marital status was coded 1 for single, 2 for married, 3 for divorced/separated, and 4 for widowed. We created three categories for employment status, which include permanent appointment (coded as 1), temporary appointment (coded as 2), and casual/contract (coded as 3). Work experience (i.e., length of years of teaching/working as a staff in the university) was categorized into < 5 years (coded as 1), 5–9 years (coded as 2), and ≥ 10 years (coded as 3). Other variables were categorized as follows: salary grade (CONTISS II grade 01–05, CONTISS II grade 06–10, CONTISS II grade 11–15, CONAUSS II Grade 01–04, and CONAUSS II Grade 05–07) [64, 65]; staff category/position was grouped into academic staff, and non-academic/clerical staff (coded as 1 and 2, respectively), and the institutional type was categorized into private university (coded as 1) and public university (coded as 2).

#### Workplace incivility

We used the 7-item Workplace Incivility Scale (WIS) developed by Cortina et al. [15] to measure experienced incivility from the supervisors and co-workers. The scale assesses the frequency of perceived incivility in the past five years. However, to minimize recall bias or ambiguity, the study participants were asked to describe their *workplace incivility experience in the last 12 months* or *academic session*. This is a shorter period than the five-year period recommended by Cortina et al. [15]. The scale comprised items that measure both direct and indirect forms of workplace aggression. Examples of items in the 7-item WIS include 'My co-workers address me in unprofessional terms, either publicly or privately,' 'My co-workers put me down or are condescending to me,' and 'My co-workers make demeaning or derogatory remarks about me.' The response format ranges from *0* (never) to *5* (daily). Next, we calculated the total WIS score for all the participants. The WIS score ranges from 0 to 35. Higher scores indicate a high level of workplace incivility experience. To assess women's supervisor and co-worker incivility experience, we dichotomized the response option into "Yes" or "No." Women answered "Yes," when their responses showed *rarely to daily* to at least one item on the WIS in the past 12 months while a never response was regarded "No." The WIS has been used in a previous study [66]. The WIS has good internal consistency reliability that ranged from 0.85 to 0.89 [67–69]. The Cronbach’s alpha reliability for the entire 7-item WIS was 0.65. The alpha coefficients for the supervisor incivility and co-worker incivility subscales were 0.50 and 0.73, respectively.

Additionally, we used the seven-item modified Workplace Incivility Scale (MWIS) developed by Blau and Andersson [70] to measure person-initiated or instigated incivility. sample questions from the MWIS include “How often have you exhibited the following behaviors in the past year to someone at work (e.g., co-worker, other employees, supervisor)? “During the past year, while employed in the current organization, have often have you made demeaning or derogatory remarks about others?” The MWIS used a 4-point Likert response format 1 = hardly ever (once every few months or less, 2 = rarely (about once a month), 3 = sometimes (at least once a week), and 4 = frequently (at least once a day). The scores range from 1 to 28, with higher scores implies much involvement in person-initiated incivility in the workplace. However, to assess women’s perpetration/involvement in instigated incivility, responses that indicated *rarely* (about once a month) to *frequently* (at least once a day) to at least one item on the MWIS were categorized as “Yes” while responses that indicated *hardly ever* to all the items on the MWIS were considered “No”. Thus, we dichotomized participants’ instigated incivility into *Yes* (coded as 1) and *No* (coded as 0). The internal consistency reliability coefficient via Cronbach’s alpha for Blau and Andersson MWIS scale was 0.81 (Additional File 1). The alpha coefficient for the combined 7-item Cortina et al. WIS and Blau and Andersson MWIS scale was 0.84 (Additional File 2).

#### Workplace bullying

The Negative Acts Questionnaire-Revised (NAQ-R) is the most used scale to evaluate workplace bullying [71–74]. The NAQ-R is a 22-item questionnaire designed to measure workplace bullying in diverse workplace settings [75, 76]. The 22 items in the NAQ-R are structured to measure bullying behaviors. The NAQ-R is a free 22-item questionnaire for use in non-commercial research projects. The NAQ-R is available in the public domain for surveys. The NAQ-R involves three different categories of negative behaviors, such as person-oriented bullying, workplace-related bullying, and physically intimidating bullying. Additionally, 12 items measure person-oriented bullying; 7 items measure work-related bullying, and 3 items measure physically intimidating bullying [75, 76]. Examples of such items include “been excluded from the social fellowship” and “exposed to exaggerated teasing and joking.” The NAQ-R has a five-point Likert scale response format to evaluate workplace bullying exposure in the past 6 months (i.e., 1 = never, 2 = occasionally, 3 = monthly, 4 = weekly, 5 = daily). We used a cut-off point of 33 on the NAQ-R to categorize the participants into two exclusive groups of bullied vs. not bullied, based on their workplace bullying exposure. Thus, participants with a score lower than 33 (< 33) are not bullied, while participants with a score greater than 33 (≥ 33) are bullied. The cut-off point has been used in a previous study [77]. The NAQ-R has good psychometric properties. [71–75]. The Cronbach’s alpha of 0.91 was obtained for the NAQ-R in this study (Additional File 3).

#### Sexual harassment (SH)

The 20-item version of the Sexual Experiences Questionnaire (SEQ) [78] was used to measure SH experiences. The SEQ is a non-proprietary instrument that is available for non-commercial research purpose. The SEQ measures three dimensions of SH, such as gender harassment, unwanted sexual attention, and sexual coercion. Participants were asked to rate the frequency of each experience on a 5-point scale that ranged from 0 (never) through 4 (many times); SEQ total scores indicate the frequency with which the participants reported experiencing SH in the university environment in the past 12 months [78, 79]. However, we dichotomized the SH experience of the participants for the prevalence analyses. We coded one or more experiences of SH as *1* (Yes)*,* while no experience/never experienced SH was coded as 0 (No). Otherwise, we used the composite score. This procedure was used by Rospenda et al. [80]. Fitzgerald et al. reported that the internal consistency coefficient for the SEQ ranged between 0.86 and 0.92, and a test–retest coefficient of 0.86 for 1 week [80]. The Cronbach's alpha coefficient for the SEQ was 0.73. The subscales' alpha coefficients were as follows: 0.77 for gender harassment; 0.72 for unwanted sexual attention; and 0.93 for sexual coercion (Additional File 4).

### Data processing and analyses

We conducted data entry, data cleaning, and coding using SPSS version 25 software (IBM Corp., Armonk, NY, USA) and analyzed with the same software. First, we conducted test of normality on the data to inform the selection of statistics used for data analyses. The normality of the continuous data was examined using the Kolmogorov–Smirnov test, and data distribution fulfilled the criteria for normality. The skewness and kurtosis were also performed. We also conducted descriptive statistics such as frequencies, means, and standard deviations (SD), and bivariate correlation analysis using Pearson’s *r* to present the information. The skewness and kurtosis values were considered appropriate for any item values if they fall within the range of + 2 or − 2 [81]. We used the Chi-squared test to examine the association between the groups (experienced/yes vs. never experienced/no) and the categorical variables. In contrast, independent samples *t*-test and one-way analysis of variance (ANOVA) were used to test mean differences in the WIS, NAQ-R, and SEQ index scores using the participants' sociodemographic variables.

Furthermore, each independent variable was fitted separately into the bivariate logistic analysis to evaluate for the degree of association with the forms of workplace GBV (incivility, bullying, and sexual harassment). We conducted bivariate logistic regression to check the crude association between the outcome variables and predictors using the forced entry method. Before the use of bivariate logistic regression, we examined multi-collinearity for all the models through the variance inflation factor (VIF) [82], and none was detected (VIF values < 5). We selected the variables with *P* < 0.05 for further exploration in the multivariable logistic regression analysis (MLR). We used the MLR analysis to identify the independently associated predictors of GBV. The staggered entry method was used for the MLR by entering first the demographic variables (age, academic qualification, and marital status), second, the work-related variables (employment status and work experience) and third, staff category was entered. We checked the goodness of fit of the final model using Hosmer and Lemeshow [83] and was found fit. The results were summarized using crude odds ratio (COR), adjusted odds ratio (AOR), and 95% confidence interval (CI). A *P*-value of 0.05 was considered as the threshold for statistical significance. Also, the study adhered to the STROBE guideline (Additional File 5).

## Results

### Descriptive statistics

A total of 301 out of 339 participants completed the survey with full information, representing an 88.8% response rate. Among the 301 that completed the questionnaires, 113 (37.5%) were academic staff, and 188 (62.5%) were non-academic staff (administrative/clerical staff). One hundred and sixty-two (53.8%) were from public universities, and 139 participants (46.2%) were from private universities. Also, 89.4% had permanent job status, 6.6% had a temporary appointment/employment status, and 4.0% had casual or contract employment status. Furthermore, 16.3% of the participants had a doctorate, 22.6% had a master's degree or its equivalent, 29.6% had a first degree, 16.6% possessed OND/NCE certificate, and 15.0% had SSCE. The mean age for participants was 40.1 years (SD = 12.9), ranging from 22 to 66 years (Table 1). Table 2 shows the means, standard deviations, and intercorrelations for all the study variables. The mean WIS score was 24.7 (SD = 7.39), and the mean NAQ-R score was 36.1 (SD = 12.9). Besides, the mean score for the SEQ was 8.30 (SD = 11.0). There was a positive moderate relationship between workplace incivility and sexual harassment (*r* = 0.36, *p* < 0.000) and workplace bullying (*r* = 0.43, *p* < 0.000). Moreover, there was a strong relationship between workplace bullying and sexual harassment (*r* = 0.76, *p* < 0.000) (Table 2).

**Table 1:** Sociodemographic characteristics of the study’s participants (*N* = 301)

Participant’s variables	Frequencies or mean (SD)	% or (range)
*Age (Years)*	40.1 (12.9)	(22–66)
18–34 years	84	27.9
35–49 years	112	37.2
≥ 50 years	105	34.9
*Academic qualification*		
Senior secondary school certificate (SSCE)	45	15.0
OND/NCE	50	16.6
B.Sc./B.Ed./B.A. or its equivalent	89	29.6
M.Sc./M.Ed./M.A. or its equivalent	68	22.6
Doctoral degree (Ph.D.)	49	16.3
*Marital status*		
Single	38	15.9
Married	217	72.1
Separated/divorced	19	6.3
Widowed	17	5.6
*Employment status*		
Permanent appointment	269	89.4
Temporal appointment	20	6.6
Casual/Contract appointment	12	4.0
*Work experience/length of service*		
< 5 years	115	38.2
5–9 years	50	16.6
** ≥ **10 years	136	45.2
**Salary grade level		
^a^CONTISS II Grade 01–05 (#360, 000—#743,475.00)	72	23.9
^b^CONTISS II Grade 06–10 (#873, 551—#2,734,592.00)	61	20.3
^c^CONTISS Grade 11–15 (#2,127, 402—#5,874,755.00)	55	18.3
^d^CONUASS II Grade 01–04 (#1,478,046—#3,125,980.00)	65	21.6
^e^CONUASS II Grade 05–07 (#3,428,047—#6,664,214.00)	48	15.9
*Staff category*		
Non-academic staff	188	62.5
Academic staff	113	37.5
*Type of institution*		
Private university	139	46.2
Public university	162	53.8

OND, Ordinary National Diploma; NCE, National Certificate of Examination; B.Sc., Bachelor of Science; B.Ed., Bachelor of Education; B.A., Bachelor of Arts; M.Sc., Master of Science; M.Ed., Master of Education; M.A., Master of Arts; CONTISS II, The Consolidated Tertiary Institutions Salary Structure II; CONUASS II, The Consolidated University Academic Staff Salary Structure II

**Salary grade, Private universities in Nigeria do not use the CONTISS II and CONUASS II. However, the salary structures for academic and non-academic staff are similar to these templates

^a^CONTISS II, Annual gross salary from step 01–15

^b^CONTISS II, Annual gross salary from step 01–11

^c^CONTISS II, Annual gross salary from step 01–09

^d^CONUASS II, Annual gross salary from step 01–09

^e^CONUASS II, Annual gross salary from step 01–09

**Table 2:** Descriptive statistics and intercorrelations among study variables

Variables	Overall WIS	Supervisor incivility	Co-worker incivility	Instigated incivility	Overall SH	Gender harassment	Unwanted SA	Sexual coercion	Overall WPB	Personal bullying	Work bullying	Physical bullying	Skewness	Kurtosis
1	–	.86**	.82**	.94**	.36**	.28**	.35**	.36**	.43**	.43**	.33**	.52**	.63	− .42
2		–	.66**	.70**	.45**	.37**	.45**	.44**	.45**	.44**	.33**	.52**	.61	− .81
3				.63**	.33**	.26**	.32**	.33**	.39**	.39**	.29**	.43**	.86	.08
4				–	.26**	.20**	.24**	.26**	.76**	.34**	.28**	.46**	.82	− .04
5					–	.89**	.92**	.92**	.72**	.79**	.56**	.61**	1.54	1.83
6						–	.70**	.78**	.67**	.72**	.58**	.52**	1.30	.91
7							–	.76**	.70**	.72**	.48**	.51**	2.81	1.08
8								–	–	.73**	.47**	.65**	1.52	1.20
9									–	.69**	.85**	.61**	.78	.85
10										–	.96**	.68**	.74	.77
											–	.76**	1.09	.98
*M*	24.7	6.06	6.55	11.6	8.30	3.21	2.95	2.14	36.1	20.6	10.7	4.96	–	–
*SD*	7..39	2.00	2.03	4.28	11.0	3.89	4.65	3.56	12.9	7.43	4.39	2.31	–	–

M, mean; SD, standard deviation; SH, sexual harassment; SA, sexual attention; WPB, workplace bullying

### Prevalence of workplace incivility, bullying, and sexual harassment

A total of 63.8% of respondents had experienced at least one form of workplace incivility during the previous session (i.e., past 12 months). In detail, 67.4% experienced supervisor incivility, 58.8% experienced coworker incivility and 52.8% experienced instigated incivility. Also, a total of 53.5% of participants had experienced at least one form of WPB. Concerning types of WPB, 47.5% of the participants initiated personal bullying, 62.5% experienced work-related bullying and 42.2% experienced physical bullying. Also, 40.5% of the women experienced sexual harassment (SH). Regarding other of forms of SH, 36.5% experienced gender harassment, 25.6% experienced unwanted sexual attention and 26.6% experienced sexual coercion (Table 3). There was a significant difference in the NAQ-R scores [*F*_(2, 298)_ = 7.663, *η*^2^ = 0.05, *p* = 0.001] among the participants of different age groups. Besides, there was a significant difference in the bullying status-bullied vs. not bullied, [χ^2^ (2) = 11.362, *p* = 0.003] among participants of different age groups. In addition, participants of diverse age groups differed significantly in their SH experience-harassed vs. never harassed [χ^2^ (2) = 7.118, *p* = 0.028]. There were significant differences in the WIS scores [*F*_(4, 296)_ = 7.593, *η*^2^ = 0.10, *p* < 0.0001], NAQ-R scores [*F*_(4, 296)_ = 3.160, *η*^2^ = 0.04, *p* = 0.014], and SEQ scores [*F*_(4, 296)_ = 3.781, *η*^2^ = 0.05, *p* = 0.005] among the participants in terms of academic qualification groups. Also, there were significant differences in the WIS scores [*F*_(2, 298)_ = 4.880, *η*^2^ = 0.03, *p* = 0.008] among women in terms of employment status. Furthermore, there were significant differences in the WIS scores [*F*_(2, 298)_ = 30.835, *η*^2^ = 0.17, *p* < 0.0001], NAQ-R scores [*F*_(2, 298)_ = 21.971, *η*^2^ = 0.13, *p* < 0.0001], and SEQ scores [*F*_(2, 298)_ = 11.423, *η*^2^ = 0.07, *p* < 0.0001] among the participants in terms of work experience. Women differed significantly in their WIS scores [*F*_(4, 296)_ = 5.560, *η*^2^ = 0.07, *p* < 0.0001], NAQ-R scores [*F*_(4, 296)_ = 3.214, *η*^2^ = 0.04, *p* = 0.013], and SEQ scores [*F*_(4, 296)_ = 3.214, *η*^2^ = 0.04, *p* = 0.031]. Moreover, there was a significant difference in WIS scores of female academic and non-academic staff [*t* (299) = -2.874, *η*^2^ = 0.03, *p* = 0.004]. In addition, female academic and non-academic staff differed significantly in their workplace incivility experience-yes vs. no [χ^2^ (1) = 6.036, *p* = 0.014], and SH experience-harassed vs. never harassed [χ^2^ (1) = 6.115, *p* = 0.013] (Tables 4, 5).

**Table 3:** Prevalence of workplace incivility, bullying and sexual harassment among university women

Variables	*n*	%
*Workplace incivility*	192	63.8
Supervisor incivility	203	67.4
Co-worker incivility	177	58.8
Instigated incivility	159	52.8
Workplace bullying	161	53.5
Personal bullying	143	47.5
Work-related bullying	188	62.5
Physical bullying	127	42.2
Sexual harassment	122	40.5
Gender harassment	110	36.5
Unwanted SA	77	25.6
Sexual coercion	80	26.6

*n* represents only the proportion of women that experienced forms of GBV

**Table 4:** Prevalence of workplace incivility, bullying and sexual harassment of women by demographic factors

Variable	Total sample *n*	Workplace incivility			Workplace bullying			Sexual harassment		
		Mean (SD)	Exp *n* (%)	Never *n* (%)	Mean (SD)	Bullied *n* (%)	Not bullied *n* (%)	Mean (SD)	Harassed *n *(%)	Never harassed *n* (%)
*Age (Years)*										
18–34 years	84	23.9 (6.48)	60 (71.4)	24 (28.6)	32.4 (15.7)	39 (46.4)	45 (53.6)	11.1 (1.21)	27 (32.1)	57 (67.9)
35–49 years	112	24.6 (8.15)	67 (59.8)	45 (40.2)	39.5 (12.2)	74 (66.1)	38 (33.9)	10.1 (0.96)	56 (50.0)	56 (50.0)
≥ 50 years	105	23.9 (7.28)	65 (61.9)	40 (38.1)	35.6 (10.3)	48 (45.7)	57 (54.3)	11.9 (1.16)	39 (37.1)	66 (62.9)
*t* (df), χ^2^ (df), *F* (df)		*F* (2, 298) = 0.307	χ^2^ (2) = 0.218		*F* (2, 298) = 7.663	χ^2^ (2) = 11.362		*F* (2, 298) = 0.710	χ^2^ (2) = 7.118	
*P*-value		0.736	0.218		0.001	0.003		0.492	0.028	
*η*^2^		.002	–	–	.05	–	–	.004	–	–
*Academic qualification*										
SSCE	45	22.5 (6.89)	25 (55.6)	20 (44.4)	31.1 (10.5)	24 (53.3)	21 (46.7)	4.9 (8.64)	9 (20.0)	36 (80.0)
OND/NCE	50	27.3 (7.55)	39 (78.0)	11 (22.0)	33.8 (7.99)	26 (52.0)	24 (48.0)	5.1 (6.20)	14 (28.0)	36 (72.0)
B.Sc./B.Ed./B.A./equiv	89	24.3 (7.91)	53 (59.6)	36 (40.4)	37.2 (15.6)	44 (49.4)	45 (50.6)	9.1 (12.4)	35 (39.3)	54 (60.7)
M.Sc./M.Ed./M.A./equiv	68	21.1 (5.50)	34 (50.0)	34 (50.0)	38.3 (13.5)	36 (52.9)	32 (47.1)	9.5 (10.7)	36 (52.9)	32 (47.1)
Doctoral degree (PhD)	49	26.6 (7.18)	41 (83.7)	8 (16.3)	38.2 (11.9)	31 (63.3)	18 (36.7)	11.6 (13.1)	28 (57.1)	21 (42.9)
*t* (df), χ^2^ (df), *F* (df)		*F* (4, 296) = 7.593	χ^2^ (4) = 20.369		*F* (4, 296) = 3.160	χ^2^ (2) = 2.523		*F* (4, 296) = 3.781	χ^2^ (2) = 21.135	
*P*-value		< 0.0001	< 0.0001		0.014	0.641		0.005	< 0.0001	
*η*^2^		.10	–	–	.04	–	–	.05	–	–
*Marital status*										
Single	38	24.7 (8.18)	34 (70.8)	14 (29.2)	32.1 (8.66)	24 (50.0)	24 (50.0)	6.8 (7.64)	18 (37.5)	30 (62.5)
Married	217	24.1 (7.34)	136(62.7)	81 (37.3)	37.3 (13.4)	122(56.2)	95 (43.8)	8.5 (11.3)	89 (41.0)	128(59.0)
Separated/divorced	19	24.3 (7.58)	11 (57.9)	8 (42.1)	32.2 (15.6)	5 (26.3)	14 (73.7)	7.1 (13.3)	5 (26.3)	14 (73.7)
Widowed	17	23.6 (6.06)	11 (64.7)	6 (35.3)	37.1 (11.9)	10 (58.8)	7 (41.2)	10.9 (12.5)	10 (58.8)	7 (41.2)
*t* (df), χ^2^ (df), *F* (df)		*F* (3, 297) = 0.109	χ^2^ (3) = 1.440		*F* (3, 297) = 2.760	χ^2^ (3) = 6.720		*F* (3, 297) = 0.723	χ^2^ (3) = 4.157	
*P*-value		0.955	0.696		0.042	0.081		0.539	0.245	
*η*^2^		.001	–	–	.03	–	–	.003	–	–
*Employment status*										
Permanent appointment	269	23.8 (7.34)	166(61.7)	103(38.3)	35.8 (13.4)	138(51.3)	131(48.7)	7.8 (11.3)	96 (35.7)	173(64.3)
Temporal appointment	20	25.8 (4.88)	17 (85.0)	3 (15.0)	37.4 (8.92)	14 (70.0)	6 (30.0)	12.9 (5.91)	17 (85.0)	3 (15.0)
Casual/Contract	12	30.2 (9.60)	9 (75.0)	3 (25.0)	41.0 (7.27)	9 (75.0)	3 (25.0)	12.3 (7.43)	3 (25.0)	9 (75.0)
*t* (df), χ^2^ (df), *F* (df)		*F* (2, 298) = 4.880	χ^2^ (2) = 5.052		*F* (2, 298) = 1.005	χ^2^ (2) = 4.941		*F* (2, 298) = 2.883	χ^2^ (2) = 24.941	
*P*-value		0.008	0.080		0.367	0.085		0.058	< 0.000	
*η*^2^		.03	–	–	.006	–	–	.02		
*Work experience*										
< 5 years	115	20.9 (6.11)	55 (47.8)	60 (52.2)	30.8 (9.73)	53 (46.1)	62 (53.9)	4.6 (6.78)	32 (27.8)	83 (72.2)
5–9 years	50	22.8 (6.56)	27 (54.0)	23 (46.0)	35.4 (8.64)	23 (46.0)	27 (54.0)	9.9 (8.82)	29 (58.0)	21 (42.0)
** ≥ **10 years	136	27.5 (7.32)	110(80.9)	26 (19.1)	40.9 (14.8)	85 (62.5)	51 (37.5)	10.9 (13.5)	61 (44.9)	75 (55.1)
*t* (df), χ^2^ (df), *F* (df)		*F* (2, 298) = 30.835	χ^2^ (2) = 31.963		*F* (2, 298) = 21.971	χ^2^ (2) = 8.099		*F* (2, 298) = 11.423	χ^2^ (2) = 15.086	
*P*-value		< 0.000	< 0.000		< 0.000	0.017		< 0.000	0.001	
*η*^2^		.17	–	–	.13	–	–	.07	–	–

OND, Ordinary National Diploma; NCE, National Certificate of Examination; B.Sc., Bachelor of Science; B.Ed., Bachelor of Education; B.A., Bachelor of Arts; M.Sc., Master of Science; M.Ed., Master of Education; M.A., Master of Arts; equiv., Equivalent; CONTISS II, The Consolidated Tertiary Institutions Salary Structure II; CONUASS II, The Consolidated University Academic Staff Salary Structure II

***p < .0001; ** *p* < .001; * *p* < .05

^a^CONTISS II, Annual gross salary from step 01–15

^b^CONTISS II, Annual gross salary from step 01–11

^c^CONTISS II, Annual gross salary from step 01–09

^d^CONUASS II, Annual gross salary from step 01–09

^e^CONUASS II, Annual gross salary from step 01–09; χ^2^ (df), Pearson’s chi-square and degree of freedom; *F*(df), *F*-ratio and degrees of freedom; *t* (df), independent-samples t-test and degree of freedom; *η*^2^, Partial Eta squared

**Table 5:** Prevalence of workplace incivility, bullying and sexual harassment and female staff by demographic factors

Variable	Total sample *n*	Workplace incivility			Workplace bullying			Sexual harassment		
		Mean (SD)	Yes *n* (%)	No *n *(%)	Mean (SD)	Bullied *n* (%)	Not bullied *n *(%)	Mean (SD)	Harassed *n* (%)	Never harassed *n* (%)
*Salary grade level*										
^a^CONTISS II Grade 01–05	72	21.6 (7.24)	33 (45.8)	39 (54.2)	34.0 (12.4)	33 (45.8)	39 (54.2)	6.6(10.28)	22 (30.6)	50 (69.4)
^b^CONTISS II Grade 06–10	61	25.7 (6.44)	46 (75.4)	15 (24.6)	40.4 (13.6)	41 (67.2)	20 (32.8)	10.9(13.62)	29 (47.5)	32 (52.5)
^c^CONTISS II Grade 11–15	55	22.6 (7.08)	31 (56.4)	24 (43.6)	32.9 (14.1)	23 (41.8)	32 (58.2)	5.9 (9.58)	15 (27.3)	40 (72.7)
^d^CONUASSII Grade 01–04	65	26.6 (6.63)	53 (81.5)	12 (18.5)	36.1 (9.23)	38 (58.5)	27 (41.5)	10.3 (9.91)	38 (58.5)	27 (41.5)
^e^CONUASSII Grade 05–07	48	24.6 (8.69)	29 (60.4)	19 (39.6)	37.7 (14.9)	26 (54.2)	22 (45.8)	8.1(10.69)	18 (37.5)	30 (62.5)
*t* (df), χ^2^ (df), *F* (df)		*F* (4, 296) = 5.560	χ^2^ (4) = 24.030		*F* (4, 296) = 3.214	χ^2^ (4) = 9.981		*F* (4, 296) = 3.214	χ^2^ (4) = 17.080	
*P*-value		0.000	0.000		0.013	0.041		0.031	0.002	
*η*^2^		.07	–	–	.04	–	–	.04	–	–
*Staff category*										
Non-academic staff	188	23.2 (7.13)	110(58.5)	78 (41.5)	35.8 (13.6)	97 (51.6)	91 (48.4)	7.7 (11.45)	66 (35.1)	122(64.9)
Academic staff	113	25.7 (7.60)	82 (72.6)	31 (27.4)	36.8 (11.9)	64 (56.6)	49 (43.4)	9.4 (10.21)	56 (49.6)	57 (50.4)
*t* (df), χ^2^ (df), *F* (df)		*t* (299) = − 2.874	χ^2^ (1) = 6.036		*t* (299) = − 0.656	χ^2^ (1) = 0.721		*t* (299) = − 0.656	χ^2^ (1) = 6.115	
*P*-value		0.004	0.014		0.512	0.396		0.201	0.013	
*η*^2^		.03	–	–	.001	–	–	.001	–	–
*Type of institution*										
Private university	139	24.1 (7.42)	86 (61.9)	53 (38.1)	35.9 (13.7)	75 (54.0)	64 (46.0)	7.5 (10.57)	50 (36.0)	89 (64.0)
Public university	162	24.3 (7.40)	106(65.4)	56 (34.6)	36.3 (12.4)	86 (53.1)	76 (46.9)	8.9 (11.36)	72 (44.4)	90 (55.6)
*t* (df), χ^2^ (df), *F* (df)		*t* (299) = − 0.817	χ^2^ (1) = 0.411		*t* (299) = − 0.284	χ^2^ (1) = 0.023		*t* (299) = − 1.171	χ^2^ (1) = 2.228	
*P*-value		0.851	0.522		0.777	0.880		0.243	0.135	
*η*^2^		.002	–	–	.002	–	–	.001	–	–

OND, Ordinary National Diploma; NCE, National Certificate of Examination; B.Sc., Bachelor of Science; B.Ed., Bachelor of Education; B.A., Bachelor of Arts; M.Sc., Master of Science; M.Ed., Master of Education; M.A., Master of Arts; equiv., Equivalent; CONTISS II, The Consolidated Tertiary Institutions Salary Structure II; CONUASS II, The Consolidated University Academic Staff Salary Structure II

***p < .0001; ** *p* < .001; * *p* < .05

^a^CONTISS II, Annual gross salary from step 01–15

^b^CONTISS II, Annual gross salary from step 01–11

^c^CONTISS II, Annual gross salary from step 01–09

^d^CONUASS II, Annual gross salary from step 01–09

^e^CONUASS II, Annual gross salary from step 01–09; χ^2^ (df), Pearson’s chi-square and degree of freedom; *F*(df), *F*-ratio and degrees of freedom; *t* (df), independent-samples t-test and degree of freedom; *η*^2^, Partial Eta squared

### Workplace incivility, bullying, sexual harassment, and associated factors

Table 6 presents the results of the analyses to examine workplace incivility, bullying, sexual harassment among female university staff, and associated factors. In both the bivariate and multivariable logistic regressions, we entered workplace incivility, bullying, and sexual harassment into the models as dependent variables, being aged ≥ 50 years, having a doctoral degree (Ph.D.), having temporal and contract appointments, having a work experience of ≥ 10 years, being on CONUASSII Grade 01–04, and being an academic staff were associated with workplace incivility experience among female staff. Furthermore, being aged 35–49 years and ≥ 50 years, having OND/NCE and first degree, being separated/divorced, having temporal and contract appointments, having work experience of 5–9 years, and ≥ 10 years were associated with workplace bullying among female staff. Also, having a doctoral degree (Ph.D.), having temporal and contract appointments, having a work experience of ≥ 10 years, and being an academic staff were associated with sexual harassment of female university staff.

**Table 6:** Binary logistic regression of associations between workplace incivility, bullying, sexual harassment, and sociodemographic factors of participants

Variables	Workplace incivility		Workplace bullying		Sexual harassment	*P*-value
	COR (95% CI)	*P*-value	COR (95% CI)	*P*-value	COR (95% CI)	
*Age*						
18–34 years	1.00		1.00		1.00	
35–49 years	0.15 (0.06, 0.41)	0.154	2.51 (1.16, 5.42)	0.019	1.26 (0.57, 2.80)	0.575
≥ 50 years	0.05 (0.01, 0.15)	0.045	0.39 (0.16, 0.94)	0.036	0.51 (0.20, 1.27)	0.148
*Academic qualification*						
SSCE	1.00		1.00		1.00	
OND/NCE	1.85 (0.61, 5.62)	0.280	0.32 (0.12, 0.89)	0.029	0.32 (0.09, 1.05)	0.060
B.Sc./B.Ed./B.A./equiv	2.02 (0.79, 5.13)	0.141	0.32 (0.13, 0.80)	0.015	1.64 (0.59, 4.56)	0.342
M.Sc./M.Ed./M.A./equiv	0.86 (0.32, 2.32)	0.771	0.53 (0.21, 1.31)	0.169	2.78 (0.99, 7.80)	0.052
Doctoral degree (PhD)	8.23 (1.99, 34.0)	0.004	1.11 (0.41, 2.99)	0.837	3.58 (1.24, 10.36)	0.019
*Marital status*						
Married	1.00		1.00		1.00	
Single	1.45 (0.73, 2.86)	0.288	0.78 (0.42, 1.46)	0.434	0.86 (0.45, 1.64)	0.654
Separated/divorced	0.82 (0.32, 2.12)	0.681	0.29 (0.10, 0.80)	0.018	0.51 (0.18, 1.48)	0.216
Widowed	1.10 (0.40, 3.07)	0.867	1.11 (0.41,1.03)	0.835	2.06 (0.75, 5.60)	0.159
*Employment status*						
Permanent appointment	1.00		1.00		1.00	
Temporal appointment	6.86 (1.46, 32.20)	0.015	7.76 (2.24, 26.83)	0.001	91.86 (14.38, 586.9)	< 0.000
Casual/Contract	19.4 (3.01, 125.5)	0.002	29.56 (4.51,193.9)	< 0.000	75.53 (7.35, 775.19)	< 0.000
*Work experience*						
< 5 years	1.00		1.00		1.00	
5–9 years	1.23 (0.43, 3.50)	0.700	0.33 (0.12, 0.89)	0.029	1.08 (0.38, 3.13)	0.885
** ≥ **10 years	23.35(8.17,66.68)	< 0.000	3.73 (1.76, 7.91)	0.001	3.96 (1.85, 8.46)	< 0.000
*Salary grade level*						
^a^CONTISS II Grade 01–05	1.00		1.00		1.00	
^b^CONTISS II Grade 06–10	2.55 (0.94, 6.97)	0.067	1.11 (0.46, 2.65)	0.822	1.71 (0.67, 4.31)	0.260
^c^CONTISS II Grade 11–15	1.38 (0.61, 3.17)	0.442	0.69 (0.31, 1.53)	0.363	0.59 (0.24, 1.47)	0.261
^d^CONUASSII Grade 01–04	10.47 (3.14, 34.9)	< 0.000	1.33 (0.52, 3.42)	0.558	2.63 (0.98, 7.10)	0.055
^e^CONUASSII Grade 05–07	1.45 (0.52, 4.08)	0.482	1.11 (0.43, 1.83)	0.830	0.87 (0.30, 2.52)	0.802
*Staff category*						
Non-academic staff	1.00		1.00		1.00	
Academic staff	1.88 (1.13, 3.11)	0.015	1.23 (0.77, 1.96)	0.396	1.82 (1.13, 2.92)	0.014
*Type of institution*						
Private university	1.00		1.00		1.00	
Public university	1.17 (0.73, 1.87)	0.522	0.87 (0.42, 1.83)	0.716	1.19 (0.52, 2.71)	0.687

OND, Ordinary National Diploma; NCE, National Certificate of Examination; B.Sc., Bachelor of Science; B.Ed., Bachelor of Education; B.A., Bachelor of Arts; M.Sc., Master of Science; M.Ed., Master of Education; M.A., Master of Arts; equiv., Equivalent; CONTISS II, The Consolidated Tertiary Institutions Salary Structure II; CONUASS II, The Consolidated University Academic Staff Salary Structure II

***p < .0001; ** *p* < .001; * *p* < .05

^a^CONTISS II, Annual gross salary from step 01–15

^b^CONTISS II, Annual gross salary from step 01–11

^c^CONTISS II, Annual gross salary from step 01–09

^d^CONUASS II, Annual gross salary from step 01–09

^e^CONUASS II, Annual gross salary from step 01–09; COR, Crude Odds Ratio; CI, Confidence Interval; 1.00, reference group

In the multivariable logistic regression model, being aged 35–49 years (AOR 0.15; 95% CI (0.06, 0.40) and ≥ 50 years (AOR 0.04; 95% CI (0.01, 0.14) were associated with workplace incivility among female staff. Female staff with doctoral degree had higher odds to experience workplace incivility compared to female staff with SSCE (AOR 8.32, 95% CI (2.01, 34.38). Women on temporal and casual/contract appointments were 7 times (AOR 6.99, 95% CI (1.48, 32.94) and 20 times (AOR 19.9, 95% CI (3.10, 128.4), respectively more likely than women with a permanent appointment to experience uncivil behaviors. Also, women with a work experience of ≥ 10 years had higher odds to experience incivility from supervisors, and co-workers compared to women with less than 5 years’ experience (AOR 23.36, 95% CI (8.19, 66.7) (Table 7). Next, women having a CONTISS II Grade 06–10 (AOR 2.73, 95% CI (1.03, 7.22) and CONUASS II Grade 01–04 (AOR 9.14, 95% CI (3.08, 27.08) had higher odds to experience workplace incivility compared to women on CONTISS II Grade 01–05.

**Table 7:** Multivariable logistic regression of associations between workplace incivility, bullying, sexual harassment, demographic and work-related characteristics of participants

*Variables	Workplace incivility		Workplace bullying		Sexual harassment	*P*-value
	AOR (95% CI)	*P*-value	AOR (95% CI)	*P*-value	AOR (95% CI)	
*Age*						
18–34 years	1.00		1.00		1.00	
35–49 years	0.15 (0.06, 0.40)	< 0.000	2.50 (1.16, 5.40)	0.020	1.25 (0.56, 2.78)	0.582
≥ 50 years	0.04 (0.01, 0.14)	< 0.000	0.39 (0.16, 0.94)	0.035	0.51 (0.20, 1.27)	0.148
*Academic qualification*						
SSCE	1.00		1.00		1.00	
OND/NCE	1.86 (0.61, 5.67)	0.273	0.32 (0.12, 0.89)	0.030	0.31 (0.09, 1.05)	0.059
B.Sc./B.Ed./B.A./equiv	2.02 (0.80, 5.13)	0.140	0.32 (0.13, 0.80)	0.015	1.64 (0.59, 4.53)	0.344
M.Sc./M.Ed./M.A./equiv	0.88 (0.33, 2.35)	0.791	0.53 (0.21, 1.32)	0.171	2.78 (0.99, 7.76)	0.052
Doctoral degree (PhD)	8.32 (2.01, 34.38)	0.003	1.11 (0.41, 2.99)	0.837	3.57 (1.24, 10.34)	0.019
*Marital status*						
Married	1.00		1.00		1.00	
Single	1.51 (0.53, 4.26)	0.438	0.36 (0.14, 0.88)	0.025	0.19 (0.06, 0.58)	0.003
Separated/divorced	0.71 (0.22, 2.28)	0.569	0.27 (0.08, 0.88)	0.029	0.74 (0.24, 2.34)	0.609
Widowed	1.63 (0.43, 6.12)	0.473	1.00 (0.33, 3.09)	0.995	1.67 (0.48, 5.77)	0.419
*Employment status*						
Permanent appointment	1.00		1.00		1.00	
Temporal appointment	6.99 (1.48, 32.94)	0.014	7.79 (2.26, 26.91)	0.001	91.26 (14.27, 583.4)	< 0.000
Casual/Contract	19.9 (3.10, 128.4)	0.002	29.93(4.57, 196.2)	< 0.000	73.81 (7.26, 750.78)	< 0.000
*Work experience*						
< 5 years	1.00		1.00		1.00	
5–9 years	1.22 (0.43, 3.49)	0.705	0.33 (0.12, 0.89)	0.029	1.08 (0.37, 3.12)	0.889
** ≥ **10 years	23.36 (8.19, 66.7)	< 0.000	3.71 (1.75, 7.86)	0.001	3.94 (1.85, 8.42)	< 0.000
*Salary grade level*						
^a^CONTISS II Grade 01–05	1.00		1.00		1.00	
^b^CONTISS II Grade 06–10	2.73 (1.03, 7.22)	0.043	1.15 (0.49, 2.69)	0.747	1.63 (0.66, 3.99)	0.288
^c^CONTISS II Grade 11–15	1.40 (0.61, 3.18)	0.429	0.70 (0.32, 1.55)	0.377	0.59 (0.24, 1.45)	0.249
^d^CONUASSII Grade 01–04	9.14 (3.08, 27.08)	< 0.000	1.22 (0.53, 2.18)	0.639	2.92 (1.25, 6.84)	0.014
^e^CONUASSII Grade 05–07	1.26 (0.52, 3.08)	0.611	1.02 (0.45, 2.33)	0.965	0.97 (0.38, 2.46)	0.948

OND, Ordinary National Diploma; NCE, National Certificate of Examination; B.Sc., Bachelor of Science; B.Ed., Bachelor of Education; B.A., Bachelor of Arts; M.Sc., Master of Science; M.Ed., Master of Education; M.A., Master of Arts; equiv., Equivalent; CONTISS II, The Consolidated Tertiary Institutions Salary Structure II; CONUASS II, The Consolidated University Academic Staff Salary Structure II; aCONTISS II, Annual gross salary from step 01–15; bCONTISS II, Annual gross salary from step 01–11; cCONTISS II, Annual gross salary from step 01–09; dCONUASS II, Annual gross salary from step 01–09; eCONUASS II, Annual gross salary from step 01–09; AOR, adjusted Odds Ratio; CI, Confidence Interval; 1.00, reference group; *Variables, only variables with *p* ≤ 0.05 were entered into the multivariable logistic regression, staff category and type of institution were not included in the model

***p < .0001; ** *p* < .001; * *p* < .05

Additionally, women aged 35–49 years had higher odds to be bullied compared to those aged 18–34 years (AOR 2.50, 95% (1.16, 5.40). However, being ≥ 50 years (AOR 0.39, 95% (0.16, 0.94) reduced the odds of being bullied in the workplace compared to being aged 18–34 years.

Having OND/NCE (AOR 0.32, 95% CI (0.12, 0.89) and a first degree (AOR 0.32 95% CI (0.13, 0.80) reduced the odds of workplace bullying compared to female staff with SSCE. Similarly, being single (AOR 0.36, 95% CI (0.14, 0.88) and separated/divorced (AOR 0.27 95% CI (0.08, 0.88) reduced the odds of workplace bullying compared to the married female staff.

Women with temporary appointment (AOR 7.79, 95% CI (2.26, 26.91) and casual/contract appointment (AOR 29.93, 95% CI (4.57, 196.2) had higher odds to report workplace bullying compared to women with a permanent appointment/employment status. Women with 5–9 years’ work experience had lesser odds to experience workplace bullying compared to women with < 5 years’ work experience in the university (AOR 0.33; 95% CI (0.12, 0.89). Also, women with ≥ 10 years had higher odds to be bullied in the university compared to women with 5 years’ work experience (AOR 3.71; 95% CI (1.75, 7.86) (Table 7).

Furthermore, having a doctoral degree, (AOR 3.57, 95% CI (1.24, 10.34), and being single (AOR 0.19, 95% CI (0.06, 0.58) were significantly associated with sexual harassment of female staff. Women with temporary appointment (AOR 91.26, 95% CI (14.27, 583.4) and casual/contract appointment (AOR 73.81, 95% CI (7.26, 750.78), respectively had higher odds to experience sexual harassment from a supervisor, head of the department/unit, senior colleagues, or other colleagues in the workplace compared to women with SSCE. The odds of being sexually harassed were higher among female staff with ≥ 10 years’ work experience (AOR 3.94, 95% CI (1.85, 8.42) compared to those with less than 5 years' work experience. Female staff on CONUASS II Grade 01–04 had higher odds to experience sexual harassment from a supervisor, head of department/unit, senior male colleagues, or other male colleagues compared to female on CONTISS II Grade 01–05 (AOR 2.92, 95% CI (1.25, 6.84).

## Discussion

### Main findings

The study aimed to examine the prevalence of workplace GBV and associated factors among female university staff. Workplace GBV is a prevalent problem in higher educational institutions and manifested as workplace incivility, bullying, and sexual harassment. In this study, the prevalence of workplace incivility, bullying, and sexual harassment was 63.8%, 53.5%, and 40.5%, respectively. The prevalence of workplace incivility, bullying, and sexual harassment in our study is higher than the reported prevalence in a Nigerian study [9]. The high prevalence of GBV in our study could be due to many factors, including women’s reluctance to report incidents of GBV, fear of social stigma, fear of consequence such as job loss, and retribution [49, 84]. This finding is consistent with the reported prevalence of GBV in previous studies [9, 14, 15, 44, 47, 85].

Also, there was a high prevalence of sub-types of workplace incivility-supervisor, coworker, and instigated incivility in our sample. A plausible explanation for our finding could be that the university women experience persistent uncivil or discourteous behaviors while performing their duties due to high job strain and demands that characterize the university environments. Also, a poor working environment characterized by lower support from co-workers, lower levels of job insecurity, reduced job satisfaction, aggression, and low incentives for workers has been linked to a higher level of incivility from coworkers [84, 86–88]. Such workplace settings foster organizational pressures that support uncivil behaviors from supervisors, colleagues, and subordinates. The findings are consistent with prior studies [15, 19, 22–24, 27, 28] which reported that coworkers perpetrate diverse forms of incivility as a retributory or retaliatory response to recent exposure to perceived or actual uncivil or rude behaviors such as low social support from supervisors and co-workers and high job demands. Future research should focus on evidence-based preventive interventions that consider the organizational aspects implicated in the persistent occurrence of workplace incivility in Nigerian university contexts. Such intervention could reduce workplace incivility in educational environments.

This study reported a high prevalence of personal, work-related, and physical bullying among our sample. The finding could suggest a persistent and prolonged problem and dysfunctional system suggestive of an organizational culture that tolerates harmful behaviors or negative acts. Consistent with the view of Cortina et al. [15], bullying variants could be attributed to the spiraling effects of negative acts in the working environment. Our findings are consistent with previous studies [15, 16, 44, 52, 88]. The findings also imply that university management needs to create a workplace climate that mitigates the negative acts since WPB is associated with poor health outcomes [39]. Interventions that identify bullying subcultures and incorporate preventive and mitigating measures, are vital for promoting health among university employees, especially women [88].

The high prevalence of SH observed in our study could be due to a poor working environment or organizational climate that permits SH's forms by supervisors, colleagues, or subordinates. For instance, studies have suggested power imbalance (i.e., power imbalance predisposes female staff to sexual coercion) in the workplace context, the offer of bonuses and promotion in return for sexual attention are prevalent in many workplaces including the academia [47]. Our findings corroborate prior research showing that sexual harassment of women is prevalent in diverse workplaces, including academia [48, 49, 52, 79, 84]. The finding is an urgent call for well-functioning support structures for SH's victims, and active organizational structures are also essential for preventing SH in higher education. Also, creating an inclusive, structurally egalitarian workplaces that ensure power balance and equality between women and men prevents sexual harassment since women in male-dominated workplaces are at higher risk of sexual harassment [84, 86, 87]. Furthermore, from a theoretical point of view, the socioecological model offers a theoretical understanding of the diversity of SH's risk factors in higher education. Thus, future intervention studies should leverage the SEM to design appropriate interventions to address the risk factors at the individual and organizational levels.

. Our results further showed that being aged 35–49 years and ≥ 50 years and having a doctoral degree were associated with workplace incivility. The finding that having a doctoral degree is associated with workplace incivility contradicts available evidence. that shows that education serves as a buffer against rude or uncivil behaviors among women in the workplace, including academia [86, 89, 90]. The finding could suggest that possession of a higher degree does not protect women from workplace uncivil behaviours. The finding is inconsistent with previous studies [9, 86, 90].

In our study, having temporary and casual/contract employment status, and work experience of ≥ 10 years increased the odds of workplace incivility among women. Additionally, the finding could suggest that marital status does not protect against exposure to rude and discourteous acts in the workplace. This finding is inconsistent with a previous study [9] that reported being married as a protective factor against GBV. Similarly, women with temporary and casual/contract employment status may be insensitive to covert or overt uncivil behaviors towards them because of their status. In many circumstances in Nigerian workplace environments, the status and privileges that come with permanent or full-time appointments are not usually accorded temporal and casual workers. To prevent job loss, women with temporal and casual/contract appointments "endure" these behaviors possibly to secure a permanent appointment or at least secure a decent means of livelihood. The limited research on the association between GBV and employment status among women in Nigeria's tertiary education community hinders finding comparison. Nevertheless, higher education institutions can provide viable mechanisms for women regardless of their educational qualification, employment status, and work experience to identify, report, and avoid rude behaviors. Similarly, incivility victims should be provided with emotional or psychological support structures that can help them build resilience against rude behaviors [91].

Furthermore, our findings showed that older age reduced the odds of bullying among university women. A reasonable explanation for the finding may be that people’s respect for old age in many Nigerian cultures inhibits the display of aggression towards older women. Future research may further explore the protective or mediating role of advanced or older age in women’s experience of GBV in higher educational environments.

Concerning the association between having a temporary and casual/contract employment (TCCE) status and WPB, female staff with TCCE status experience WPB due to non-existence or ineffective mechanisms to deal with WPB and fear of retribution. Many victims of WPB may not have sought help because they perceive the university-oriented policy and support structures as dysfunctional. Thus, higher education institutions should provide emotional or psychological support structures that can help them build resilience against rude behaviors [91]. In general, university administrations should explore measures that support gender-related expectations about how people should be treated, which permeate countries, industries, professions, and work domains [89].

Furthermore, our findings showed that having a doctoral degree, being single and having a TCCE status, work experience of ≥ 10 years, and having a low income (CONUASS II Grade 01–04, i.e., #1, 478,046-#3,125, 980) [64, 65] were significantly associated with sexual harassment among university women. A plausible explanation for the findings could be unsafe working conditions, inactive or passive leadership, inequalities between men and women in terms of accessibility to research funding, a societal normalization of GBV, toxic academic masculine cultures, and poor economic condition [86, 92]. The findings agree with previous studies [2, 9, 13, 14, 80, 92]. Further, university administrators could adopt standard guidelines and policies that provide employees with criteria for acceptable and non-acceptable behavior regarding sexual harassment in the workplace [47]. Social support from colleagues and supervisors for a victim could help in ameliorating adverse health outcomes following SH [79]. Also, there is a need for restructuring working conditions in higher education, especially for women, challenging toxic academic masculine cultures, and implementing viable measures to eradicate men's violence against women [86]. Although women’s financial or economic condition improves overtime as they advance through the ranks in academia, women who currently earn an annual income between #1, 478,046 (i.e., equivalent of USD 3,213 at the current exchange rate of #460 per 1 USD) and #3,125, 980 (USD 6795.60) could be exposed to SH due persistent economic problems. however, research evidence on the association between women’s income and SH is mixed. Studies suggested that higher income is a protective factor and low income is a risk factor [92, 93]. Another study reported that higher income does not immune women from sexual assault or harassment [94, 95]. Nevertheless, interventions to increase university women’s access to economic opportunities such as research grants, scholarships and other financial incentives may help mitigate the incidence of SH.

### Study strengths and weaknesses

The present study offers new insights and valuable evidence on the prevalence of GBV forms and associated factors among university women, an under-studied group in health surveys in Nigerian academia. The cross-sectional nature of the present study limits the ability to draw any conclusions concerning the associated factors of GBV, and thus, causality cannot be established. Future studies that employ more robust designs such as experimental or longitudinal research methodologies may help establish causality. Another limitation of this study is the small sample size. Future research would benefit from a larger sample size. The use cut-off points on the NAQ-R for dichotomization of university women’s GBV experience may lead to overestimation or underestimation of WPB prevalence in our study. However, since the tool's psychometric properties have been established in many populations or subgroups, our findings are comparable with previous studies. This situation could potentially be addressed in future studies by adopting objective measures of WPB so that findings do not only reflect the individual’s subjective responses. The study data were also collected subjectively and retrospectively, although this method is more convenient and beneficial for surveys. However, there is the possibility of recall bias and response biases since research evidence suggests that women tend not to report SH experience for fear of stigma or retribution. Nevertheless, we used standardized anonymous scales which have potential to significantly reduce response bias due to social desirability and sensitive items. Besides, the study participants were drawn from the high educational setting (university environments). Thus, the generalizability of findings to other higher education settings such as colleges of education, monotechnics, polytechnics, and sectors may be limited. Despite these limitations, the survey reflects the current situation of GBV in many Nigerian university environments.

## Conclusion

There was a high prevalence of GBV (incivility, bullying, and sexual harassment) among university women. Interrelationship was found between women’s experience of incivility, bullying, and sexual harassment in the university environment. Women's experience of forms of GBV in the workplace was significantly associated with their age, higher academic qualification, marital status, having temporal and contract/casual appointment, and work experience of ≥ 10 years. This study's findings could inform the development of evidence-based interventions in university environments to prevent workplace GBV and its detrimental effects on women’s health. Also, such interventions should be aimed at eliminating different forms of GBV and addressing associated factors to reduce the adverse mental, physical, and social health outcomes among women. In addition, human resource policies that focus on addressing GBV in academia are imperative.

## Supplementary Information

**Additional file 1:** Reliability test results for MWIS.

**Additional file 2:** Reliability test results for WIS.

**Additional file 3:** Reliability test results for NAQ-R.

**Additional file 4:** Reliability test results for SEQ.

**Additional file 5: **STROBE Checklist.

## Data Availability

All data generated or analysed during this study are included in this published article [and its supplementary information files].

## Abbreviations

GBV: Gender-based violence
B.Sc.: Bachelor of Science
B.Ed.: Bachelor of Education
B.A.: Bachelor of Arts
NCE: National Certificate of Examination
NPC: National Population Commission
M.Sc.: Master of Science
M.Ed.: Master of Education
M.A.: Master of Arts
MLR: Multivariable logistic regression
OND: Ordinary National Diploma
SEM: Socio-ecological model
SEQ: Sexual Experiences Questionnaire
NAQ-R: Negative Acts Questionnaire-Revised
SH: Sexual harassment
WPB: Workplace bullying
WIS: Workplace incivility scale
WSH: Workplace sexual harassment
TCCE: Temporal and casual/contract employment status
LGA: Local Government Area
UwSA: Unwanted sexual attention
AOR: Adjusted odds ratio
COR: Crude odds ratio
CI: Confidence interval
IRB: Institutional Review Board
VIF: Variance inflation factor
