# Supplementary material for: Workplace gender-based violence and associated factors among university women in Enugu, South-East Nigeria: an institutional-based cross-sectional study
Source: BMC Womens Health. 2021 Mar 23;21:124. doi: 10.1186/s12905-021-01273-w (PMC7988966; doi:10.1186/s12905-021-01273-w)
Supplement: Supplementary file 5 — Additional file 5: STROBE Checklist. [file 12905_2021_1273_MOESM5_ESM.docx]

**Additional File 5.** STROBE Checklist

|  | **Item no** |  |
| --- | --- | --- |
| **Title and Abstract** | 1 | Workplace gender-based violence and associated factors among university women in Enugu, South-East Nigeria: an institutional based cross-sectional study. Title page, P1, Line 1-2.  Abstract, P2, Line 31-56; P3-Line 57-60. |
| **Introduction** |  |  |
| Background/rationale | 2 | In Nigeria, the prevalence of GBV is high. Previous studies reported that GBV is an important public health problem in Nigeria [4, 8].  For instance, a study [8] reported that about 52.1% of the women indicated that the incidence of domestic violence is high, while 63.3% had experienced domestic violence at one time or the other. In the university environment, a study [9] reported that the life-time prevalence of intimate partner violence (IPV) was 42.3% among undergraduate and postgraduate female students.  Previous studies have indicated that GBV is a predominant phenomenon in higher educational institutions [9, 12]. However, this problem is still under-studied in the educational institutions in developing nations [13]. Thus, it is possible that there is a paucity of data available on the prevalence of GBV among university women in Nigeria.  Background, paragraph 2, P3-P8, Line 62-194. |
| Objectives | 3 | This study aimed to determine whether the prevalence of incivility, bullying and sexual harassment (i.e., forms of GBV) is high among university women and examines if women's GBV experiences are associated with their personal factors and contextual variables (staff category, employment status). Next, we hypothesized that there are interrelationships among the outcomes-workplace bullying, incivility, and sexual harassment.  Background, Paragraph 2, P7-P8, Line 190-198. |
| **Methods** |  | P8, Line 199 |
| Study design | 4 | Institutional based Cross-sectional study. Methods, Paragraph 1, P8, Line 201 |
| Setting | 5 | The study was conducted in Enugu, Enugu State. Enugu state is one of the states in south-east, Nigeria. The study period covered five months from May 25 to October 30, 2019.  Methods, Paragraph 1, P8, Line 201-202. |
| Participants | 6 | The population for the study comprises 4,995 female staff in the sampled universities during the 2018/2019 academic session.  Methods, Paragraph 1, P8, Line 211-213.  The inclusion criteria include working for at least six months as a university staff, issuance of voluntary informed consent, and absence of ill health at the time of the study, and voluntary informed consent. Exclusion criteria include a work experience of less than 6 months, and refusal to participate in the study, and ill health.  Methods, Paragraph 1, P9, Line 235-238. |
| Variables | 7 | The primary outcome variables were workplace incivility, bullying and sexual harassment.  Methods (measures), Paragraph 1, P9-12, Line 240-321. |
| Data sources/  measurement | 8 | Workplace incivility. We used the 7-item Workplace Incivility Scale (WIS) developed by Cortina et al. [15] to measure experienced incivility from the supervisors and co-workers.  Workplace bullying. The NAQ-R was used to measure workplace bullying. The Negative Acts Questionnaire-Revised (NAQ-R) is the most used scale to evaluate workplace bullying [73-76]. The NAQ-R is a 22-item questionnaire designed to measure workplace bullying in diverse workplace settings [77-78]. The 22 items in the NAQ-R are structured to measure bullying behaviours.  Methods (measures), Paragraph 1, P9-12, Line 240-321. |
| Bias | 9 | To address recall bias, the participants were asked to self-report their experiences of GBV in the past 12 months. In addition, to address information bias, we used well-validated instruments for data collection. Paragraph 1, P9-12, Line 240-321. |
| Study size | 10 | We used the Leslie Kish single population proportion formula to calculate the study sample size. We assumed the prevalence of workplace incivility, bullying and sexual harassment to be 30% among female university staff with a 95% confidence level, and 5% margin of error. In addition, a 5% non-response rate was added to the initial sample size. Thus, 339 women constituted the study sample size.  $n= \frac{(Z\alpha/2)^{2}*p(1-p)}{d^{2}}$  The calculated sample size for the study was 323. Afterwards, the sample size was multiplied by 5% non-response rate (323*0.05 = 16) and was added to 323 (i.e., 323 + 16). Finally, the study sample size was determined to be 339. Methods (Sample size determination and procedure),  Paragraph 2, P8-9, Line 214-223; P9, Line 223-238. |
| Quantitative variables | 11 | To assess women’s experience of supervisor and co-worker incivility, we dichotomized the response option into “Yes” or “No”. Women answered “Yes”, when their responses indicated *rarely* to *daily to at least one item on the WIS in the past 12 months* while *a never response was regarded “No”*. Methods (Measures), Paragraph 1, P10-11, Line 265-270.  To assess women’s perpetration/involvement in instigated incivility, responses that indicated rarely (about once a month) to frequently (at least once a day) to at least one item on the MWIS were categorized as “Yes” while responses that indicated hardly ever to all the items on the MWIS were considered “No”. Thus, we dichotomized participants’ instigated incivility into Yes (coded as 1) and No (coded as 0).  Methods (Measures), Paragraph 1, P11, Line 291-298.  To facilitate categorization of participants based on their workplace bullying exposure status (bullied vs not bullied), we used a cut-off point of 33. Thus, participants with a score lower than 33 (< 33) are not bullied while participants with a score greater than 33 (≥ 33) are bullied. The cut-off point has been used in a previous study [79].  Methods (Measures), Paragraph 2, P11-12, Line 299-313.  We dichotomized the SH experience of the participants for the prevalence analyses. We coded one or more experiences of SH as 1 (Yes) while no experience/never experienced SH was coded as 0 (No). Otherwise, we used the composite score. This procedure was used by Rospenda et al. [80]. Methods (Measures), Paragraph 1, P12, Line 319-326. |
| Statistical methods | 12 | We conducted descriptive statistics such as frequencies, means and standard deviations (SD). We also conducted test of normality on the data to inform the selection of statistics used for data analyses. The normality of the continuous data was examined using the Kolmogorov-Smirnov test, and data distribution fulfilled the criteria for normality. skewness, kurtosis, and bivariate correlation analysis using Pearson’s r to present the information. The skewness and kurtosis values were considered appropriate for any item values if the fall within the range of +2 or –2 [81]. We used the Chi-squared test was used to examine the association between the groups (experienced/yes vs. never experienced/no) and the categorical variables while independent samples t-test and one-way analysis of variance (ANOVA) were used to test mean differences in the WIS, NAQ-R and SEQ index scores using the participants’ sociodemographic variables. Furthermore, each independent variable was fitted separately into the bivariate logistic analysis to evaluate for the degree of association with the forms of workplace GBV (incivility, bullying and sexual harassment). We conducted bivariate logistic regression to check the presence of the crude association between the outcome variables and predictors using the forced entry method. Prior to the use of bivariate logistic regression, we examined multi-collinearity for all the models through the variance inflation factor (VIF) [82], and none was detected (VIF values < 10). We selected the variables with P < 0.05 for further exploration in the multivariable logistic regression analysis (MLR). We used the MLR analysis to identify the independently associated predictors of GBV. We checked the goodness of fit of the final model using Hosmer and Lemeshow [83] and was found fit. The results were summarized using crude odds ratio (COR), adjusted odds ratio (AOR), and 95% confidence interval (CI). A *P*-value of 0.05 was considered as the threshold for statistical significance.  Methods (Data processing and analyses), Paragraph 1, P12-13, Line 327-351. |
| **Results** |  | P13, Line 352 |
| Participants | 13 | A total of 301 out of 339 participants completed the survey with full information, representing an 88.8% response rate (Table 1). Results section, Paragraph 1, P13, Line 353-355. |
| Descriptive data | 14 | Among the 301 that completed the questionnaires, 113 (37.5%) were academic staff and 188 (62.5%) were non-academic staff (administrative/clerical staff). One hundred and sixty-two (53.8%) were from public universities and 139 participants (46.2%) were from private universities. Also, 89.4% had permanent job status, 6.6% had temporal appointment, and 4.0% had casual or contract employment status. Furthermore, 16.3% of the participants had a doctorate degree, 22.6% had a master’s degree or its equivalent, 29.6% had a first degree, 16.6% possessed OND/NCE certificate and 15.0% had SSCE. The mean age for participants was 40.1 years (SD = 12.9), ranging from 22 to 66 years (Table 1). Table 2 shows the means, standard deviations, and intercorrelations for all the study variables. The mean WIS score was 24.7 (SD = 7.39) and the mean NAQ-R score was 36.1 (SD = 12.9). In addition, the mean score for the SEQ was 8.30 (SD = 11.0). There was a positive moderate relationship between workplace incivility and sexual harassment (*r* = .36, *p* < 0.000) and workplace bullying (*r* = .43, *p* < 0.000). Moreover, there was a strong relationship between workplace bullying and sexual harassment (*r* = .76, *p* < 0.000) (Table 2).  Results section, Paragraph 1, P13-14, Line 354-367. |
| Outcome data | 15 | A total of 63.8% of respondents had experienced at least one form of workplace incivility during the previous session (i.e., past 12 months). In detail, 67.4% experienced supervisor incivility, 58.8% experienced coworker incivility and 52.8% experienced instigated incivility. Also, a total of 53.5% of participants had experienced at least one form of WPB. Concerning types of WPB, 47.5% of the participants initiated personal bullying, 62.5% experienced work-related bullying and 42.2% experienced physical bullying. Also, 40.5% of the women experienced sexual harassment (SH). Regarding other of forms of SH, 36.5% experienced gender harassment, 25.6% experienced unwanted sexual attention and 26.6% experienced sexual coercion (Table 3).  Results section, Paragraph 2, P 20, Line 511-518, |
| Main results | 16 | The major findings showed that being aged 35-49 years (AOR 2.75; 95% CI (1.31, 5.77), and ≥ 50 years (AOR 0.40; 95% CI (0.17, 0.95) were associated with workplace incivility among female staff. Female staff with OND/NCE, first degree and master’s degree were 0.34 times (AOR 0.34, 95%CI (0.12, 0.93), 0.31 times (AOR 0.31, 95%CI (0.13, 0.77), and 0.51 times (AOR 0.51, 95%CI (0.21, 1.27), respectively less likely to experience workplace incivility compared to female staff with SSCE. Married female were 2.69 times (AOR 2.69, 95%CI (1.10, 6.57) more likely than single female staff to experience workplace incivility. Women on temporal and casual/contract appointments were 7.48 times (AOR 7.48, 95%CI (2.20, 25.50) and 29.7 times (AOR 29.70, 95%CI (4.68, 189.40), respectively more likely than women with permanent appointment to experience uncivil behaviours. Also, women with a work experience of ≥ 10 years were 3.83 times (AOR 3.83, 95%CI (1.85, 7.94) more likely than women with less than 5 years’ experience to be exposed to incivility from supervisors, and co-workers (Table 6).  Additionally, being aged 35-49 years (AOR 0.27, 95% (0.11, 0.64) and ≥ 50 years (AOR 0.55, 95% (0.12, 0.17) reduced the odds of being bullied in the workplace compared to being aged 18-34 years. However, women with doctoral degrees were 8.21 times (AOR 8.21, 95% CI (2.22, 30.40) more likely than women with SSCE to report bullying by a supervisor, head of department/unit, senior colleagues, or other colleagues. Women with temporal appointment (AOR 6.88, 95%CI (1.54, 30.71) and casual/contract appointment (AOR 12.21, 95%CI (2.00, 74.4) were 6.88 times and 12.21 times, respectively more likely than women with permanent appointment to report being bullied in the workplace. Also, women with ≥ 10 years were 23.20 times (AOR 23.20; 95% CI (8.52, 63.90) more likely than women with 5 years’ work experience to be bullied in the university. Female academic staff were 2.10 times (AOR = 2.10, 95% CI (1.11, 3.98) more likely than female non-academic staff to report bullying by a supervisor, head of department/unit, senior colleagues, or other colleagues (Table 6).  Furthermore, having a doctoral degree (AOR 4.01, 95% CI (1.44, 11.22), being married (AOR 4.10, 95% CI (1.46, 11.50), and widowed (AOR 6.01, 95% CI (1.30, 27.78) were significantly associated with sexual harassment of female staff. Women with temporal appointment (AOR 60.54, 95% CI (11.00, 333.1) and casual/contract appointment (AOR 42.05, 95% CI (5.25, 336.83), respectively were 60.54 times and 42.05 times more likely than women with SSCE to be sexually harassed by a supervisor, head of department/unit, senior colleagues, or other colleagues in the workplace. The odds of being sexually harassed were 4.18 times (AOR 4.18, 95% CI (1.99, 8.79) more likely among female staff with ≥ 10 years than those with less than 5 years’ work experience. Female academic staff were 1.82 times (AOR 1.82, 95% CI (1.04, 3.18) more likely than female non-academic staff to be sexually harassed by a supervisor, head of department/unit, senior colleagues, or other colleagues (Table 6).  Results section, Paragraphs 2-3, P22, Line 549-574; P23, Line 575-583. |
| Other analyses | 17 | N/A |
| **Discussion** |  | Discussion section  P23 Line 586-602; P24 Line 603-630; P25, Line 631-658; P26, Line 659-686; P27, Line 687-701. |
| Key results | 18 | The prevalence of workplace incivility, bullying, and sexual harassment was high among university women. Discussion (main findings), Paragraph 1, P23, Line 586-593. |
| Limitations | 19 | The cross-sectional nature of the present study limits the ability to draw any conclusions concerning the associated factors of GBV, and thus, causality cannot be established. Future studies that employ more robust designs such as experimental or longitudinal research methodologies may be helpful in establishing causality. Another limitation of this study is the small sample size. Future research would benefit from a larger sample size. The use cut-off points on the NAQ-R for dichotomization of university women’s GBV experience may lead to overestimation or underestimation of the prevalence of WPB in our study. However, since the psychometric properties of the tool have been established in many populations or subgroups, our findings are comparable with previous studies. This situation could potentially be addressed in future studies by adopting objective measures of WPB so that findings do not only reflect the individual’s subjective responses. Also, the study data were collected subjectively and retrospectively, although this method is more convenient and beneficial for surveys, however, there is the possibility of recall bias and response biases since research evidence suggests that women tend not to report SH experience for fear of stigma or retribution. In addition, the study participants were drawn from the high educational sector, thus, generalizability of findings to other sectors may be limited. Despite these limitations, the survey reflects that current situation of GBV in many Nigerian university environments.  Strengths and weaknesses, Paragraph 1, Line 703-714; P 27; P28, Line 715-723. |
| Interpretation | 20 | We provided a cautious overall interpretation of our results considering objectives, limitations, multiplicity of analyses, results from similar studies, and other relevant evidence. We also considered residual confounding due to unmeasured variables such as leadership style, job demands, work conflicts, social support, and other institutional factors.  Discussion (Main findings) P23-27. |
| Generalizability | 21 | Since the data used in the current study were from a state in southeast Nigeria, and limited to only the university women, the findings may not be generalizable to all women who are employed in colleges of education, monotechnics, polytechnics and other sectors in the country.  Strengths and weaknesses, Paragraph 1, Line 646-648, P27; Line 649-664, P28. |
| **Other information** |  | N/A |
| Funding | 22 | This research received no specific grant from any funding agency in public, commercial, or not-for-profit sectors. The study was funded by the authors.  Funding, P29, Line 749-750, |

*Note.* N/A = Not Applicable.
